# A case report of minimally invasive percutaneous ultrasound guided tuberculous iliopsoas abscess drainage in an immunocompromised patient

**DOI:** 10.1016/j.ijscr.2022.106867

**Published:** 2022-02-26

**Authors:** Allyzain Ismail, Neelam Ismail, Athar Ali, Shabbir Adamjee, Ahmed Jusabani, Zainab Fidaali

**Affiliations:** a The Aga Khan University, East Africa Medical College, Tanzania; b The Aga Khan Hospital, Dar-es-Salaam, Tanzania

**Keywords:** Psoas abscess, Tuberculosis, Interventional radiology, Minimally invasive procedure, Case report

## Abstract

**Introduction and importance:**

Iliopsoas abscess is a collection of pus that presents with nonspecific features with often delays in diagnosis however cause significant morbidity and mortality with *Mycobacterium tuberculosis* to be considered as causative agent in at risk individuals in tuberculous endemic regions. Management involves drainage and initiation of adequate antibiotics with radiological guided percutaneous approach considered the appropriate initial approach.

**Case presentation:**

50-year-old immunosuppressed presenting with left iliopsoas abscess who underwent ultrasound guided drainage and placement of pigtail catheter successfully without the need for open surgical drainage. Our experience of interventional radiology for diagnosis of causative agent and treatment in a sub-Saharan Africa.

**Clinical discussion:**

We concur with the recommendation to analyse fluid for tuberculosis in at risk individuals with minimally invasive procedures via interventional radiology as an adequate first line diagnostic and treatment option of psoas abscess. Ultrasound guided catheter placement and drainage successfully drained the abscess by day 10 similarly seen as the average duration in a case series from India.

**Conclusion:**

The importance of the role of interventional radiology in treatment for complex abdominal pathologies in sub-Saharan Africa with its ability to diagnose and treat via minimally invasive procedures at highest precision and lowest risks and complications while maintaining a high level of suspicion for tuberculosis as the underlying etiology is highlighted.

## Highlights

• Minimally invasive procedure for drainage of a tuberculous iliopsoas abscess
• Image guided diagnosis and therapy
• Interventional radiology experience in sub-Saharan Africa
• Level of suspicion of tuberculous iliopsoas abscess in at risk individuals

## 1 Introduction and importance

Iliopsoas abscess is an uncommon disease whereby there is a collection of pus in the iliopsoas muscle compartment that presents with nonspecific features hence causing delays in diagnosis however causing significant morbidity and mortality [1]. It is classified as either primary, via hematological or lymphatic spread from a distant site, or secondary abscess, via direct spread from adjacent structures [2]. In Africa majority of iliopsoas abscesses are primary with risk factors of human immunodeficiency virus (HIV) infection, diabetes, intravenous drug abuse, and renal failure [3]. Primary abscesses are most commonly due to an infection by a single organism with *Staphylococcus aureus* being the most common pathogen isolated. However, in tuberculous endemic regions *Mycobacterium tuberculosis* must be considered in at risk individuals [1], [4].

Symptoms include back pain, flank pain, inguinal swelling, limping gait, anorexia, fever and weight loss but due to its non-specific characteristics as well as mimicking other gastrointestinal, renal and musculoskeletal conditions there is often a delay in diagnosis. It has shown to have an average time of 22 days from onset of symptoms to diagnosis with more than a third of patients being diagnosed 1 month from onset [5]. Computed tomography (CT) is the imaging modality of choice which reveals an obvious hypodense area with infiltration of surrounding fat and air fluid levels seen within the lesion [6]. Contrast enhanced CT reveals enhancement of the wall. Ultrasound also is used for diagnosis but it may not show the exact extent of the disease. Management involves drainage and initiation of adequate antibiotics with a percutaneous approach under ultrasound or CT guidance as a suitable initial approach [7].

We report a case of a woman with HIV who presented with an inguinal swelling for 1 month and was found to have a tuberculous psoas abscess who underwent ultrasound guided percutaneous drainage with concurrent anti-tuberculosis management. In this case report from Aga Khan Hospital, Dar-es-Salaam, Tanzania, we describe our approach to psoas abscess highlighting importance of interventional radiology in a Sub-Saharan region that lacks this expertise to greater extent. This paper has been reported in line with the SCARE 2020 criteria [8]. This article has been registered with the Research Registry with identification number researchregistry7523 and can be found through the following hyperlink Browse the Registry - Research Registry.

## 2 Case presentation

A 50-year-old female self-presenting to the medical outpatient clinic, with known HIV positive status on Tenofovir 300 mg, Lamivudine 300 mg, and Dolutegravir 50 mg (TLD) regimen and on her fifth month of treatment for sputum positive pulmonary tuberculosis (TB) presented with a 2-month history of left inguinal swelling of gradual onset, increasing in size, painful which was radiating to the left flank, associated with low grade fevers, night sweats, general body malaise and weight loss. No history of change in bowel habit, urinary symptoms nor gastrointestinal symptoms. She otherwise had no drug allergies, no significant family history of disease, and does not smoke or drink alcohol. She had 2 prior lower segment cesarean sections. During the course of the illness had been treated with multiple antibiotic regimens which she could not recall at different centers without identification of foci of infection with no relief.

On examination she was alert, oriented, mildly pale, febrile low grade (37.8 °C), multiple hyperpigmented non-itching plaques all over her skin with stable vitals. Her abdominal examination revealed a left inguinal fluctuant swelling, 6 × 10 cm, tender, warm, not increasing in size with Valsalva maneuver with associated nearby inguinal lymphadenopathy. Rest of abdominal examination revealed no obvious mass nor hepatosplenomegaly and rest of systemic examinations were normal.

**Fig. 1:**
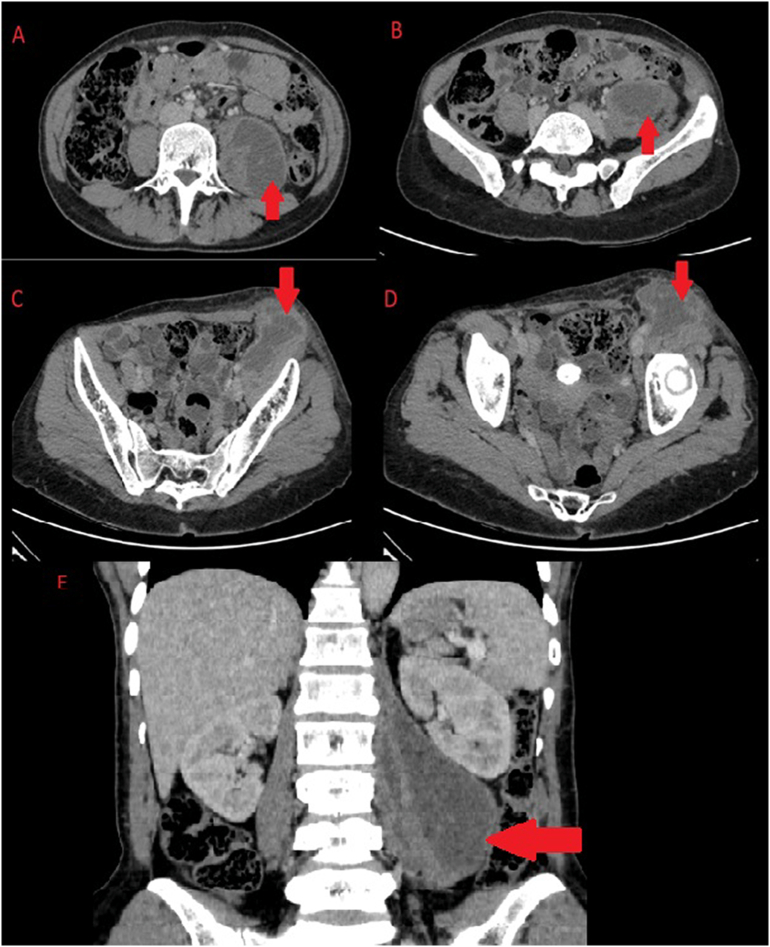
CT of abdomen. A to B - Large, well-defined smoothly marginated multiloculated lesion of fluid attenuation in left retroperitoneum involving the left psoas and iliacus muscles (Axial Images). C to D - Peripheral continuous rim enhancement in continuity with left psoas and iliacus muscle margin up to inguinal region consistent with left iliopsoas abscess extending into left inguinal region (Axial Images). E – Coronal view illustrating the iliopsoas abscess.

**Fig. 2:**
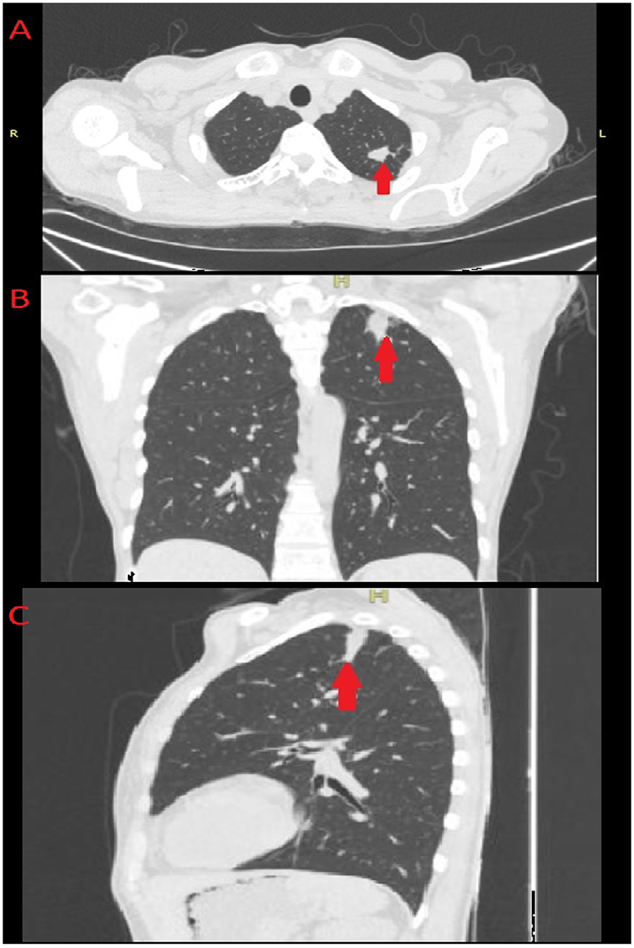
CT of chest - Non-enhancing lobulated mass located in the left upper zone posterolateral without cavitation suggestive of a differential of infective pulmonary granulomatous disease. A – Axial slice. B – Coronal view. C – Sagittal view.

The initial workup of the patient revealed a neutrophilia, microcytic hypochromic anemia with a raised inflammatory marker C-reactive protein (CRP) of 147 mg/l (normal range 0.5–5 mg/l) with a normal stool and urinalysis. In investigating for the source of infection and cause of inguinal swelling a CT scan of the chest and abdomen revealed large, well-defined multiloculated lesion of fluid attenuation in the left retroperitoneum involving the left psoas and iliac muscles with a continuous rim enhancement in continuity with left iliopsoas muscle up to inguinal region consistent with left iliopsoas abscess, along with left necrotic inguinal lymph nodes (Fig. 1). The chest scan revealed two small non-enhancing lobulated masses, located in the left upper zone posterolateral aspect, without cavitation suggestive of an infective pulmonary granuloma suspected to be tuberculous in origin (Fig. 2).

**Fig. 3:**
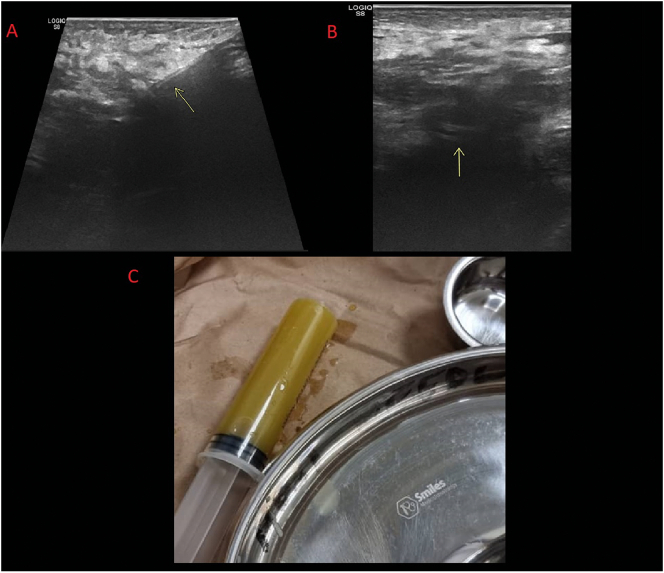
A to B – Ultrasound guided pigtail insertion into abscess. C – Initial pus aspirated.

In view of the diagnosis of psoas abscess, the patient was reviewed by a senior general surgeon and consultant radiologist and admitted for intravenous (IV) pain and antipyretic management with IV paracetamol 1 g 8 hourly and scheduled for a percutaneous ultrasound guided drainage of the abscess. Following the informed consent patient was prepared and draped under aseptic technique and a size 10 French pigtail catheter was inserted under ultrasound guidance through the left inguinal swelling which drained thick viscous pus (Fig. 3).

**Fig. 4:**
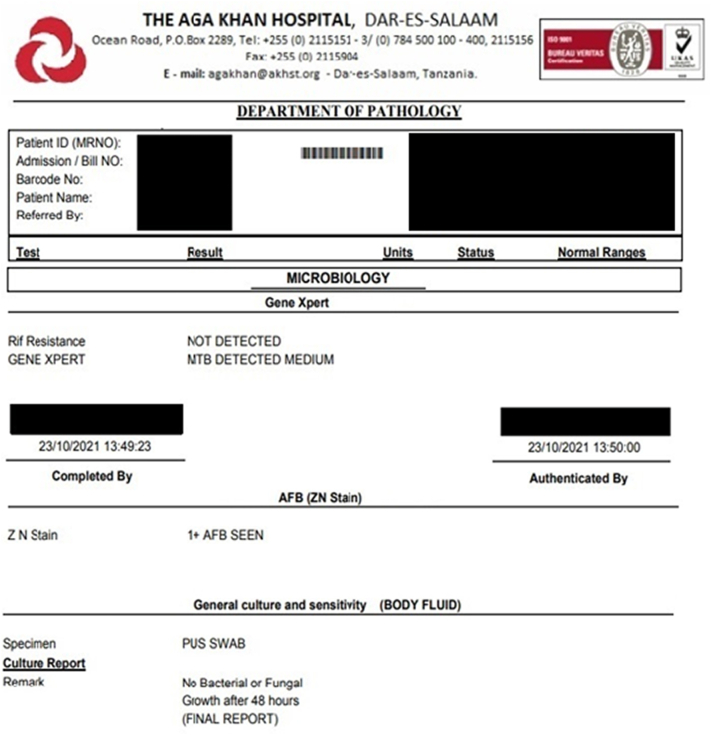
Gene-Xpert, Ziehl-Neelsen stain and culture report of pus fluid.

Post procedure patient initiated on antibiotic (IV cefepime 1 g12 hourly), pain management (IV paracetamol 1 g 8 hourly and intramuscular diclofenac 75 mg as per need basis), to continue with her antiretrovirals (TLD once daily) and antituberculosis medication (rifampicin 300 mg and isoniazid 150 mg) and for daily drain flushing with warm normal saline. Pus fluid analysis via polymerase chain reaction GENE XPERT revealed *Mycobacterium tuberculosis* without rifampicin resistance and culture and sensitivity revealed no other bacterial or fungal growth (Fig. 4). Consultation with a physician with infectious medicine background recommended to change antituberculosis regimen to rifampicin 150 mg, isoniazid 75 mg, pyrazinamide 400 mg and ethambutol 275 mg for 3 months initial phase followed by rifampicin 300 mg and isoniazid 150 mg for 6 months continuous phase.

**Fig. 5:**
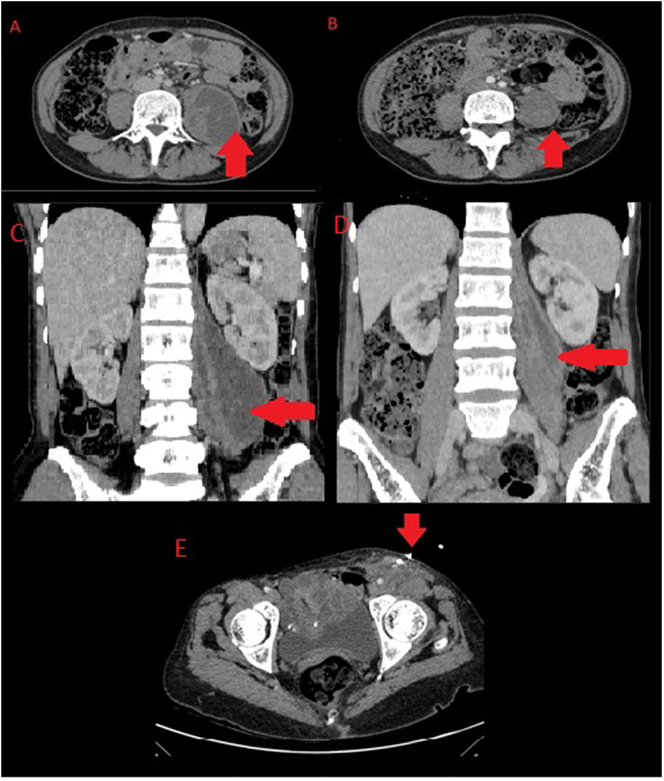
CT abdomen. A – Psoas abscess prior to drainage (Axial Image). B – Residual fluid 10 days post insertion of pigtail catheter (Axial Image). C – Psoas abscess prior to drainage (Coronal Image). D – Residual fluid 10 days post insertion of pigtail catheter (Coronal Image). E – Pigtail in situ.

Initially 900 ml drained over 24 h followed by 250 ml on day 2 and 200 ml on day 3 of same color and viscosity. Day 3 and 4 drained 50 ml and 40 ml respectively with the pus fluid becoming less viscous. At discharge on day 4 post pigtail insertion the patient's neutrophilia had normalized and CRP had dropped to 65 mg/l with pain controlled and resolution of fevers and general body malaise. Pigtail catheter was left in situ to allow for continuous drainage with daily flushing to continue as outpatient and was discharged on her antiretrovirals and antituberculosis medication with counselling on drain care. At 10 days post procedure drainage over 24 h was less than 10 ml of serous like fluid and CRP had almost normalized hence CT of the abdomen was performed revealing remarkable reduction in size of left iliopsoas abscess with surgical draining tube in situ, thus the drainage tube was removed (Fig. 5). On last follow up at outpatient surgical clinic 3 months later she was progressing well with no significant complaints, complete resolution of fevers, night sweats while gaining appetite and weight adequately with adherence towards her antiretrovirals and antituberculosis medication.

## 3 Discussion

Primary psoas abscess tend to present with chronic or subacute symptoms with vague nonspecific symptoms hence often leading to late diagnosis as seen with our patient who presented to our facility 2 months later after receiving multiple courses of antibiotics at other centers without identification of foci of infection [5]. Most TB psoas abscesses are secondary to TB sacroiliitis however the history of pulmonary TB in our patient raised our suspicion hence the decision to test the drained fluid [9]. Level of suspicion should be raised in TB endemic countries such as ours whereby recent statistics from world health organization placed Tanzania among the top 30 most affected countries with TB with around 253 per 100,000 population falling ill of which 28% were HIV positive [10]. Raised inflammatory markers of CRP of more than 10 mg/l and Erythrocyte Sedimentation Rate of more than 100 mm/h are sensitive tests for presence of TB however lack in specificity thus play a role in raising suspicion for TB as well as to monitor serial levels to monitor progress of disease in response to treatment [11], [12]. Our patient was immunosuppressed, living in a TB endemic region presenting with elevated CRP levels hence, we concur with the recommendation to analyse drained fluid for TB in at risk suspected individuals as our results was evident for rifampicin susceptible *Mycobacterium tuberculosis* only with no growth of any other bacteria.

The evolution of minimally invasive procedures with interventional radiology, the first line treatment of psoas abscess has now shifted from open surgical drainage towards percutaneous drainage via ultrasound or CT guidance. Advancements in imaging techniques and personnel expertise has helped favour this approach as it leads to lower morbidity and mortality with a shorter hospital stay, quicker wound recovery and less complications as compared to the conventional open surgical approach by avoiding general anesthesia and surgical stress [13]. Concerns however have been raised on its role in patients presenting with severe sepsis, thick pus collections and for those abscesses due to underlying conditions requiring surgical intervention such as diverticulitis. Risk of recurrence, dislocation and obstruction of the drain have been seen in a study from Turkey requiring placement of another percutaneous drain which would eventually lead to successful drainage without the need for open surgical drainage nor debridement [14].

In a study from India on tuberculous psoas abscess delineated with pre procedure CT which were drained via ultrasound guided percutaneous drainage with placement of a drainage catheter successful drainage was achieved, on average within 10 days, in 84.6% of cases with no significant complications noted [15]. Needle aspiration alone without placement of a catheter was seen to be ineffective and showed high levels of recurrence [13]. As with our case ultrasound guided catheter placement and drainage was left for 10 days at which on the tenth day minimal fluid was draining over a 24-hour period with repeat CT scan showing successful drainage of abscess and follow up 3 months later revealing no residual collection.

## 4 Conclusion

There are few case reports on role of interventional radiology for abdominal pathologies in sub-Saharan Africa due to lack of resources and expertise. In our setting of a TB endemic region a report from 2018 of just 60 radiologists from a population of almost 58 million highlights the burden on the health care system having to opt for open invasive procedures when percutaneous approaches have shown to be successful. We hope through our successful experience to encourage the use of minimally invasive percutaneous drainage of psoas abscess while maintaining a high level of suspicion for TB as the underlying etiology.

## Abbreviations

CRP: C-reactive protein
CT: computed tomography
HIV: human immunodeficiency virus
IV: intravenous
TLD: Tenofovir 300 mg, Lamivudine 300 mg, and Dolutegravir 50 mg

## Patient's perspective

I had visited multiple centers and got antibiotics which did not help me but once I was told of my condition I was surprised. However not needing surgery was a major relief and staying with a drain for 10 days was uncomfortable but at the end of it I felt much better avoiding major surgery.

## Sources of funding

This research did not receive any specific grant from funding agencies in the public, commercial, or not-for-profit sectors.

## Author contribution

A.I: study conception, production of initial manuscript, collection of data.

N.I: Revision of the manuscript, proofreading.

A.A: Revision of the manuscript, proofreading.

S.A: Production of initial manuscript, collection of data.

A.J: Revision of the manuscript, proofreading.

Z.F: Study conception, revision of the manuscript, proofreading.

## Ethical approval

Case study is exempt from ethical approval in my institution.

## Consent

Written informed consent was obtained from the patient for publication of this case report and accompanying images. A copy of the written consent is available for review by the Editor-in-Chief of this journal on request.

## Registration of research studies

1. Name of the registry: RESEARCH REGISTRY.
2. Unique identifying number or registration ID: researchregistry7523.
3. Hyperlink to your specific registration (must be publicly accessible and will be checked): Browse the Registry - Research Registry.

## Guarantor

Dr. Zainab Fidaali, zainab.fidaali@akhst.org Guarantor.

Consultant radiologist, the Aga Khan Hospital, Dar-es-Salaam, Tanzania.

P. O. Box 2289, Barack Obama Drive, Dar es Salaam, Tanzania.

## Provenance and peer review

Not commissioned, externally peer-reviewed.

## Declaration of competing interest

None.
